# Preconception care of women with diabetes: a review of current guideline recommendations

**DOI:** 10.1186/1472-6874-10-5

**Published:** 2010-01-31

**Authors:** Maimunah Mahmud, Danielle Mazza

**Affiliations:** 1 Department of General Practice, School of Primary Health Care, Faculty of Medicine, Nursing and Health Sciences, Monash University, Victoria, Australia; 2 Jinjang Health Clinic, Kuala Lumpur Health Department, Kuala Lumpur, Malaysia; 3 Department of General Practice, School of Primary Health Care, Faculty of Medicine, Nursing and Health Sciences, Monash University, Victoria, Australia

## Abstract

**Background:**

The prevalence of type 2 diabetes mellitus (T2DM) continues to rise worldwide. More women from developing countries who are in the reproductive age group have diabetes resulting in more pregnancies complicated by T2DM, and placing both mother and foetus at higher risk. Management of these risks is best achieved through comprehensive preconception care and glycaemic control, both prior to, and during pregnancy. The aim of this review was to compare the quality and content of current guidelines concerned with the preconception care of women with diabetes and to develop a summary of recommendations to assist in the management of diabetic women contemplating pregnancy.

**Methods:**

Relevant clinical guidelines were identified through a search of several databases (MEDLINE, SCOPUS and The Cochrane Library) and relevant websites. Five guidelines were identified. Each guideline was assessed for quality using the AGREE instrument. Guideline recommendations were extracted, compared and contrasted.

**Results:**

All guidelines were assessed as being of high quality and strongly recommended for use in practice. All were consistent in counselling about the risk of congenital malformation related to uncontrolled blood sugar preconceptionally, ensuring adequate contraception until glycaemic control is achieved, use of HBA1C to monitor metabolic control, when to commence insulin and switching from ACE inhibitors to other antihypertensives. Major differences were in the targets recommended for optimal metabolic control and opinion regarding the usage of metformin as an adjunct or alternative treatment before or during pregnancy.

**Conclusions:**

International guidelines for the care of women with diabetes who are contemplating pregnancy are consistent in their recommendations; however some are more comprehensive than others. Having established current standards for the preconception care of diabetic women, there is now a need to focus on guideline implementation through an examination of the barriers and enablers to successful implementation, and the applicability of the recommendations in the local setting.

## Background

The prevalence of type 2 diabetes mellitus (T2DM) continues to rise worldwide [1,2], with population based studies reporting increases in Malaysia [3], the UK [4], the USA [5], Europe [6] and Australia [7]. In developed countries, more than half of all people with diabetes are older than 65 years, and only 8% of adults with diabetes are younger than 44. In contrast, three quarters of people affected by diabetes in developing countries are under 65 years old, and 25% of all adults with diabetes are younger than 44 [8]. Thus more women of reproductive age in developing countries have diabetes, resulting in an increased number of pregnancies complicated by T2DM [9-11] placing both mother and foetus at higher risk of morbidity and mortality [12].

Diabetes in pregnancy is associated with higher rates of miscarriage, pre-eclampsia, preterm labour and higher rates of fetal malformation [13]; neural tube defect, urinary tract disorder, macrosomia, birth injury, and perinatal mortality [14]. These risks can be minimised by optimal glycaemic control, both prior to and throughout the pregnancy [15,16], and is best achieved through comprehensive preconception care where other issues such as genetic risks, health status, reproductive history, exposure to environment toxins, immunisation and lifestyle risk factors can also be addressed through a multidisciplinary approach in community based management of diabetes before and during pregnancy [17,18].

Two previous guideline comparisons for the care of pregnant women (including preconception care) with diabetes exist [19,20]. The first was a comparison of international and New Zealand guidelines. This study reported a variation in recommendations for folate supplementation with the dose ranging from 0.4 mg to 5 mg and while the authors comment that the other preconceptional recommendations were broadly similar, the guidelines lacked contraceptive advice, specification of HbA1C targets and recommendations regarding medication review. The second review was conducted by the Centers for Disease Control (CDC); however, this review was restricted to guidelines available in the United States. The aim of this current review was to compare the quality and content of current national and international guidelines that are concerned with the preconception care of women with diabetes, and to develop a summary of recommendations to assist in the management of diabetic women contemplating pregnancy.

## Methods

A systematic search was conducted of databases (Ovid Medline, the Cochrane Library, SCOPUS), guideline websites (clinical guidelines on Medical Journal of Australia website, Scottish Intercollegiate Guidelines Network, National Institute for Health and Clinical Evidence, New Zealand Guidelines Group, National Guideline Clearinghouse (US)) and other relevant websites (the American Diabetes Association, the Australasian Diabetes in Pregnancy Society, the Royal Australian College of General Practitioners and the Royal Australian and New Zealand College of Obstetricians and Gynaecologists). Search terms included: preconception care, pre conception care, preconception health, pre conception health, preconception, pre conception, prepregnancy care, pre pregnancy care, type 2 diabetes, diabetes mellitus and guidelines.

Our inclusion criteria were national and international English language guidelines published from 2001 to May 2009. We excluded those that were local or hospital based. Five guidelines that matched the criteria were identified [21-25]. Each guideline was scored for guideline quality by two independent appraisers using the AGREE instrument [26]. Differences were discussed and resolved. We compared and contrasted the guideline recommendations under the headings of: management approach, evaluation of previous medical and obstetric history, evaluation and treatment of diabetic complications, review of current medication, assessment of metabolic control, blood glucose management, folate supplementation, preconception counselling, contraindications to pregnancy and thyroid screening (Table 1). From this, we developed a summary of preconception care recommendations for use by practising physicians.

**Table 1:** A comparison of international guidelines recommendation for preconception care among diabetes

	ADA 2009	ADA 2004	NICE 2008	SIGN 2001	ADIPS 2005
**Management by multidisciplinary team**	✓	✓		✓	✓

**Complete preconception evaluation of medical and obstetric history**		✓		✓	

**Evaluation and treatment of diabetic complications**					

retinopathy	✓	✓	✓	✓	✓

nephropathy	✓	✓	✓		✓

neuropathy	✓	✓			✓

cardiovascular disease	✓	✓			✓

hypertension		✓		✓ target BP should be < 140/80	

**Medication review**					

Review all current medication	✓	✓		✓	✓

Stop Angiotensin-Converting Enzyme (ACE) inhibitors	✓	✓	✓	✓	✓

Stop Angiotensin-II Receptor Blockers (ARB)	✓		✓		✓

Stop statins	✓		✓		✓

Stop diuretics		✓			

Stop β-blockers		✓	✓	✓	

**Assessment of metabolic control**					

Measure	Use HbA1C	Use HbA1C	Use HbA1C	Use HbA1C	Use HbA1C

Frequency of testing		1-2 monthly			

Target level	< 7%	Up to 1% above normal value, lower if possible	< 6.1%	optimised HbA1C	< 7%

**Blood glucose management**					

Self monitoring targets		Before meals 4.4-6.1 mmol/l, 2 hours after meal <8.6 mmol/l		Between 4 and 7 mmol/L	

Educate regarding hypoglycaemia awareness and management			✓		✓

Prescribe insulin to achieve target blood glucose levels	✓	✓	✓	✓	✓

Use metformin as an adjunct or alternative	✓		✓		✓

**Folate supplementation**					

Commencement			preconception	preconception	Preconception

Dose			5 mg/day	5 mg/day	5 mg/day

Duration			until 12 weeks gestation	until 12 weeks gestation	

**Preconception Counselling**					

	should be routinely incorporated into diabetic care			is essential	

Advise of risk of malformation with poor metabolic control and unplanned pregnancy	✓	✓	✓	✓	✓

Advise use of effective contraception until good glucose control is achieved before conception	✓	✓	✓	✓	✓

Inform woman about how DM affects pregnancy and how pregnancy affects DM		✓	✓		

Encourage smoking cessation					✓

Encourage reduction in alcohol intake					✓

Provide dietary advice			✓	✓	

Advice about weight reduction			Aim for a BMI < 27		Encourage weight management and exercise

**Contraindications to pregnancy**			HbAIC > 10%		Creatinine > 0.2 mmol/L

**Measure thyroid function in women with Type 1 DM**		✓			✓

## Results

Of the five guidelines identified, two were from the American Diabetes Association (ADA) (ADA 2009 [21] and ADA 2004 [22]). The ADA 2009 is the generic guideline for medical care in diabetes with a specific chapter on pre-conception care, whereas the ADA 2004 guideline is focussed on preconception care of women with diabetes. The remaining guidelines were produced by the National Institute for Health and Clinical Excellence (NICE) (NICE 2008 [23]), the Scottish Intercollegiate Guideline Network (SIGN) (SIGN 2001 [24]), and the Australasian Diabetes in Pregnancy Society (ADIPS) (ADIPS 2005[25]).

### Comparison of guideline quality

All guidelines were rated as being of high quality and strongly recommended for use in practice using the AGREE instrument. Scores for individual guidelines using the AGREE instrument are given in Table 2.

**Table 2:** AGREE final scores for identified guidelines

	ADA 2009	ADA 2004	NICE 2008	SIGN 2001	ADIPS 2005
**Scope and Purpose**	94%	100%	100%	100%	94%

**Stakeholder involvement**	96%	50%	50%	75%	50%

**Rigour of development**	71%	71%	100%	100%	71%

**Clarity and presentation**	96%	100%	100%	100%	83%

**Applicability**	100%	100%	100%	67%	67%

**Editorial independence**	50%	50%	50%	50%	50%

### Comparison of guideline recommendations

#### Management approach and evaluation of previous medical and obstetric history

All the practice guidelines agree that diabetic women contemplating pregnancy should be seen by a multidisciplinary team; however this was not specifically mentioned by the NICE guidelines. Both the ADA 2009 and the SIGN guidelines recommend undertaking a complete preconception evaluation of the women's medical and obstetric history.

#### Evaluation and treatment of diabetic complications

Evaluating and treating diabetic retinopathy is recommended by all the guidelines and nephropathy by all except SIGN. The ADA and ADIPS guidelines also recommend an assessment of neuropathy and undertaking a cardiovascular assessment prior to conception.

Specifically assessing the women for hypertension is only recommended by ADA 2004 and SIGN with the latter nominating a target blood pressure (BP) of < 140/80 for women with diabetic nephropathy.

#### Review of current medication

Undertaking a medication review before a diabetic woman gets pregnant is recommended by all the guidelines except NICE. While stopping Angiotensin-Converting Enzyme (ACE) inhibitors is mentioned by all, some guidelines also recommend stopping statins [21,23,25] and angiotensin-II receptor antagonists (ARB) [21,23]. The ADA 2004 also recommends stopping diuretics and β-blockers.

#### Assessment of metabolic control and blood glucose management

Monitoring metabolic control using HbA1C and achieving a target of < 7% is recommended by all guidelines. NICE however, sets a lower target of < 6.1%. Blood glucose self monitoring is another important recommendation by most of the guidelines apart from ADA 2009 or the ADIPS 2005 guidelines. The ADA 2004 defines a target pre-meal blood glucose level of between 4.4-6.1 mmol/L and 2 hours after meals <8.6 mmol/L. All guidelines, except for the ADA 2009, confirm that blood sugar levels should be maintained as normal as possible whilst avoiding hypoglycaemia. Both the NICE and ADIPS guidelines advocate teaching the patient and partner about hypoglycaemia awareness and management [23,25].

All guidelines recommend prescribing insulin preconceptionally to achieve target levels of blood glucose. Use of metformin as an adjunct or alternative for diabetic treatment preconceptionally when insulin treatment is refused or a patient develops resistance, is recommended by the more recent guidelines [21,23,25].

#### Folate supplementation

Folate supplementation with a daily dose of 5 mg is recommended by three of the guidelines for those planning to become pregnant up until 12 weeks gestation. ADIPS does not mention the duration of treatment. Both ADA 2009 and ADA 2004 fail to mention the importance of folate supplementation.

#### Preconception counselling

The routine incorporation of preconception counselling into diabetic clinic visits for all women of child-bearing potential is recommended by ADA 2009. All guidelines recommend that diabetic women should receive counselling about the effective use of contraception in order to plan pregnancies, and the risk of malformation with poor metabolic control and unplanned pregnancy. Informing the patient about how Diabetes Mellitus (DM) affects pregnancy and how pregnancy affects DM is recommended by two guidelines [22,23]. Other important preconception issues like smoking cessation and reducing alcohol intake is only recommended by ADIPS 2005. ADIPS recommends weight management and exercise as general pre-pregnancy advice and NICE recommends weight reduction for women with a BMI >27 kg/m^2^. Provision of dietary advice such as consuming a diet with high levels of complex carbohydrates, soluble fibre and vitamins, and reduced levels of saturated fats is also recommended [23,24].

#### Contraindications to pregnancy

The NICE guideline states that an HbA1C > 10% is a contraindication to pregnancy. In contrast ADIPS suggests that impaired renal function as measured by a serum creatinine > 0.2 mmol/L should be a contraindication to pregnancy.

#### Thyroid screening

Thyroid function screening is recommended by ADA 2004 and ADIPS for women with Type 1 diabetes but not T2DM.

A summary of preconception care recommendations for diabetic women is provided in Table 3.

**Table 3:** Summary of recommendations for preconception care among diabetic women

Utilise a multidisciplinary team to manage preconception care issues
Members of the team may include an obstetrician, endocrinologist, family physician, diabetic educator and dietician

**Complete a full medical and obstetric evaluation in the preconception period to assess risks**

**Evaluate and treat diabetic complications including:**

Retinopathy (pre-existing retinopathy may progress rapidly in pregnancy and should be treated first before pregnancy)

Nephropathy (patients with pre-existing microalbuminuria are more likely to develop pre-eclampsia)

Neuropathy

Cardiovascular disease

Hypertension

**Review all current medication use including complementary medication and change the following to a form of therapy which has less risk:**

Angiotensin-Converting Enzyme (ACE) inhibitors

Angoitensin-II Receptor Blockers (ARB)

Statins

Diuretics

β-blockers

**Assess level of metabolic control**

Measure HbA1C monthly until control is achieved

HbA1C should remain below 7% (1% above normal value), lower if possible

**Blood glucose management**

Undertake blood glucose self monitoring with targets pre-meal of 4.4-6.1 mmol/l and 2 hour after meal of < 8.6 mmol/l

Maintain blood sugar within normal range without hypoglycaemia

Educate on hypoglycaemia awareness and management

Insulin should be prescribed to achieve target blood glucose levels

Use metformin as an adjunct or alternative

**Counselling**

Commence folate supplementation 5 mg daily pre-conceptually until 12 weeks gestation to prevent neural tube defects

Inform about risk of miscarriage, congenital malformation and perinatal mortality with poor metabolic control and unplanned pregnancy

Inform about how DM affects pregnancy and how pregnancy affects DM

Use effective contraception until target blood glucose control is achieved before conception

Encourage smoking cessation and reduction in alcohol intake

Encourage regular exercise and management of weight to achieve a BMI < 27

Encourage diet with high levels of complex carbohydrates, soluble fibre and vitamins and reduced levels of saturated fats

**Contraindications to pregnancy**

HbA1C >10%

Impaired renal function, creatinine > 0.2 mmol/L (increased risk of progression to dialysis during pregnancy)

**Measure thyroid function in women with Type 1 Diabetes**

## Discussion

A key finding of this review is that all the guidelines selected for comparison are of high quality and highly recommended for use in practice. Secondly, the guidelines share consistency regarding counselling about the risk of congenital malformation related to uncontrolled blood sugar preconceptionally and the use of effective contraception until good blood sugar control is achieved. Despite general agreement in all categories of recommendations, there is a lack of specific practice based recommendations in certain areas. These include which type of contraception is most suitable and how long before conception folate supplementation should be started. While medication review and advice to stop ACE inhibitors is consistently given by all the guidelines, alternative antihypertensives that are safe in pregnancy are only suggested by ADIPS, however, there is considerable evidence supporting the use of methyldopa, oxprenolol, clonidine, labetalol, prazosin and nifedipine in pregnancy [27].

Key differences observed in the recommendations are target levels of HbA1C to be achieved prior to pregnancy. Aiming the HbA1C level at <1% above the upper limit of normal (generally <7%) is recommended [21,22,24,25,28] in preparation for pregnancy. However, there is evidence of better outcomes if the HbA1C is maintained within the normal range or lower during early pregnancy [29,30]. In view of this, NICE has chosen the lower value at HbA1C as < 6.1% as a target.

Blood glucose self monitoring is recommended by all the guidelines; however the target levels are again different. When considering the two suggested targets, it may be more helpful to women to use the more specific one (pre-meal at between 4.4-6.1 mmol/l and 2 hour after meal < 8.6 mmol/l [22]).

Deterioration of diabetic complications is another concern in pregnancy among diabetic women [31]. As recommended by all guidelines, evaluating and treating diabetic complications are important actions to undertake before conception. However, not all guidelines outline all the possible complications that need to be evaluated. This may lead to inconsistency in practice amongst physicians.

The other obvious differences in the recommendations are in the use of oral hypoglycaemic agents. Though the safety of currently available oral antidiabetic agents (metformin and glyburide) during pregnancy looks promising, the complete safety and efficacy profile during the full term of pregnancy has not yet been established [32-34]. As such it is recommended to be used as an alternative by ADA 2009, ADIPs and NICE in situations such as refusal of the patient to use insulin or insulin resistance, when the likely benefits from improved glycaemic control outweigh the potential for harm [35].

Counselling is a major component of preconception care. The feasibility of routine incorporation into all practice visits need to be considered. Diabetic women are more likely to be engaged with health system and therefore there are more opportunities for delivery of preventive care. On top of specific counselling for diabetic women with pregnancy, general preconception care [36] for women should not be forgotten and should include depression screening, genetic and family history risk assessment, immunisation, smoking cessation advice, advice regarding reducing alcohol intake, weight management and exercise.

A strength of this review is that all guidelines reviewed in this series are of high quality and highly recommended to be used as practice guidelines according to a respected and validated assessment tool- the AGREE instrument [26]. The limitation of this review is the omission of non-English language guidelines.

## Conclusions

International guidelines for the care of women with diabetes who are contemplating pregnancy are consistent in their recommendations; however, some are more comprehensive than others. Having established current recommendations for the preconception care of diabetic women, there is now a need to focus on guideline implementation. More work is needed to look at the applicability of the recommendations in the local setting, and to specifically examine what barriers and enabling factors exist to ensure successful implementation.

## Competing interests

The authors declare that they have no competing interests.

## Authors' contributions

MM undertook the study and drafted the manuscript. DM conceived the study and reviewed the manuscript. Both authors read and approved the final manuscript.

## Pre-publication history

The pre-publication history for this paper can be accessed here:

http://www.biomedcentral.com/1472-6874/10/5/prepub

## References

[B1] Bringer J Fontaine P Detournay B Nachit-Ouinekh F Brami G Eschwege E Prevalence of diagnosed type 2 diabetes mellitus in the French general population: The INSTANT study Diabetes & Metabolism 2009 35 1 25 31 10.1016/j.diabet.2008.06.004 19046913

[B2] Wild S Roglic G Green A Sicree R King H Global prevalence of diabetes: estimates for the year 2000 and projections for 2030 Diabetes Care 2004 27 5 1047 1053 10.2337/diacare.27.5.1047 15111519

[B3] Institute for Public Health (IPH) 2008 The Third National Health and Morbidity Survey (NHMS 111) 2006 2 Malaysia: Ministry of Health 253

[B4] Fleming DM Cross KW Barley MA Recent changes in the prevalence of diseases presenting for health care Br J Gen Pract 2005 55 589-595 PMC1463227 16105366

[B5] Leibson CL O'Brien PC Atkinson E Palumbo PJ Melton LJ Relative contributions of incidence and survival to increasing prevalence of adult-onset diabetes mellitus: a population based study Am J Epidemio 1997 146 12 22 10.1093/oxfordjournals.aje.a009187 9215219

[B6] Passa P Diabetes trends in Europe Diabetes Metab/Res Rev 2002 18 S3 S8 10.1002/dmrr.276 12324978

[B7] Dunstan DW Zimmet PZ Welborn TA de Courten MP Cameron AJ Sicree RA Dwyer T Colagiuri S Jolley D Knuiman M The Rising Prevalence of Diabetes and Impaired Glucose Tolerance Diabetes Care 2002 25 829 834 10.2337/diacare.25.5.829 11978676

[B8] World Health Organization, International Diabetes Federation Diabetes action now. An initiative of the World Health Organization and the International Diabetes Federation Geneva 2004 http://www.who.int/diabetes/actionnow/en/DANbooklet.pdf

[B9] McElduff A Ross GP Lagstrom JA Champion B Flack JR Lau SM Moses RG Seneratne S McLean M Cheung NW Pregestational diabetes and pregnancy: an Australian experience Diabetes Care 2005 28 5 1260 1261 10.2337/diacare.28.5.1260 15855607

[B10] Cheung NW McElduff A Ross GP Type 2 diabetes in pregnancy: a wolf in sheep's clothing Australian & New Zealand Journal of Obstetrics & Gynaecology 2005 45 6 479 483 10.1111/j.1479-828X.2005.00480.x 16401211

[B11] Bell R Bailey K Cresswell T Hawthorne G Critchley J Lewis-Barned N No Diabet Pregnancy Survey S Trends in prevalence and outcomes of pregnancy in women with pre-existing type I and type II diabetes BJOG-an International Journal of Obstetrics and Gynaecology 2008 115 4 445 452 10.1111/j.1471-0528.2007.01644.x 18271881

[B12] Feig DS Palda VA Type 2 diabetes in pregnancy: a growing concern Lancet 2002 359 9318 1690 1692 10.1016/S0140-6736(02)08599-9 12020549

[B13] Ray JG Vermeulen MJ Meier C Wyatt PR Risk of congenital anomalies detected during antenatal serum screening in women with pregestational diabetes QJM-An International Journal of Medicine 2004 97 10 651 653 15367735 10.1093/qjmed/hch107

[B14] The Diabetes Control and Complications Trial Research Group Pregnancy outcomes in the Diabetes Control and Complications Trial Am J Obstet Gynecol 1996 174 4 1343 1353 10.1016/S0002-9378(96)70683-X 8623868

[B15] Kitzmiller JL Buchanan TA Kjos S Combs CA Ratner RE Pre-conception care of diabetes, congenital malformations, and spontaneous abortions Diabetes Care 1996 19 5 514 541 8732721 10.2337/diacare.19.5.514

[B16] Ray JG O'Brien TE Chan WS Preconception care and the risk of congenital anomalies in the offspring of women with diabetes mellitus: a meta-analysis QJM-An International Journal of Medicine 2001 94 8 435 444 11493721 10.1093/qjmed/94.8.435

[B17] Hadden DR Alexander A McCance DR Traub AI No Ireland Diabet G, Ulster Obstet S Obstetric and diabetic care for pregnancy in diabetic women: 10 years outcome analysis, 1985-1995 Diabetic Medicine 2001 18 7 546 553 10.1046/j.1464-5491.2001.00520.x 11553183

[B18] Hadden D Traub A Outcome of pregnancy in women with insulin dependent diabetes - Centralisation of care leads to better outcome British Medical Journal 1998 316 7130 550 550 9501724 PMC2665665

[B19] Cutchie WA Cheung NW Simmons D Comparison of international and New Zealand guidelines for the care of pregnant women with diabetes Diabetic Medicine 2006 23 5 460 468 10.1111/j.1464-5491.2006.01850.x 16681554

[B20] Mulholland C Njoroge T Mersereau P Williams J Comparison of guidelines available in the united states for diagnosis and management of diabetes before, during, and after pregnancy Journal of Womens Health 2007 16 6 790 801 10.1089/jwh.2007.CDC7 17678449

[B21] American Diabetes Association Standards of medical care in diabetes-2009 Diabetes Care 2009 32 Suppl 1 S13 61 19118286 10.2337/dc09-S013 PMC2613589

[B22] American Diabetes Association Preconception care of women with diabetes Diabetes Care 2004 27 Suppl 1 S76 78 14693933 10.2337/diacare.27.2007.s76

[B23] NICE Clinical Guideline 63. Diabetes in pregnancy: management of diabetes and its complication from pre-conception to the postnatal period http://www.nice.org.uk/nicemedia/pdf/CG063Guidance.pdf

[B24] SIGN Guideline 55: Management of Diabetes, Section 8: Management of Diabetes in pregnancy http://www.sign.ac.uk/guidelines/fulltext/55/section8.html

[B25] The Australian Diabetes in Pregnancy Society Consensus Guidelines for the Management of Patients with Type 1 and Type 2 Diabetes in Relation to Pregnancy Medical Journal of Australia 2005 1 30 16201957

[B26] AGREE Collaboration Development and validation of an international appraisal instrument for assessing the quality of clinical practice guidelines: the AGREE project Qual Saf Health Care 2003 12 1 18 23 10.1136/qhc.12.1.18 12571340 PMC1743672

[B27] Brown MA Hague WM Higgins J Lowe S McCowan L Oats J Peek MJ Rowan JA Walters BNJ The detection, investigation and management of hypertension in pregnancy: full consensus statement Australian & New Zealand Journal of Obstetrics & Gynaecology 2000 40 2 139 155 10.1111/j.1479-828X.2000.tb01137.x 10925900

[B28] McIntyre HD Flack JR Consensus statement on diabetes control in preparation for pregnancy - In reply Medical Journal of Australia 2005 182 3 141 142 15698367 10.5694/j.1326-5377.2005.tb06626.x

[B29] Suhonen L Hiilesmaa V Teramo K Glycaemic control during early pregnancy and fetal malformations in women with Type I diabetes mellitus Diabetologia 2000 43 1 79 82 10.1007/s001250050010 10663219

[B30] Evers IN de Valk HW Visser GHA Risk of complications of pregnancy in women with type 1 diabetes: nationwide prospective study in the Netherlands British Medical Journal 2004 328 7445 915 918A 10.1136/bmj.38043.583160.EE 15066886 PMC390158

[B31] Kaaja R Vascular complications in diabetic pregnancy Thrombosis Research 2009 123 S1 S3 10.1016/S0049-3848(09)70001-5 19217462

[B32] Chitayat L Jovanovic L Hod M New modalities in the treatment of pregnancies complicated by diabetes: Drugs and devices Seminars in Fetal & Neonatal Medicine 2009 14 2 72 76 10.1016/j.siny.2008.09.001 19097953

[B33] Klieger C Pollex E Koren G Treating the mother-protecting the unborn: The safety of hypoglycemic drugs in pregnancy Journal of Maternal-Fetal & Neonatal Medicine 2008 21 3 191 196 10.1080/14767050801929653 18297574

[B34] Ekpebegh CO Coetzee EJ Merwe L van der Levitt NS A 10-year retrospective analysis of pregnancy outcome in pregestational Type 2 diabetes: comparison of insulin and oral glucose-lowering agents Diabetic Medicine 2007 24 3 253 258 10.1111/j.1464-5491.2007.02053.x 17305787

[B35] Simmons D Walters BNJ Rowan JA McIntyre HD Metformin therapy and diabetes in pregnancy Medical Journal of Australia 2004 180 9 462 464 15115425

[B36] Atrash HK Johnson K Adams M Cordero JF Howse J Preconception care for improving perinatal outcomes: The time to act Maternal and Child Health Journal 2006 10 5 S3 S11 10.1007/s10995-006-0100-4 16773452 PMC1592246

